# Efficacy of Bacterial Cellulose as a Carrier of BMP-2 for Bone Regeneration in a Rabbit Frontal Sinus Model

**DOI:** 10.3390/ma12152489

**Published:** 2019-08-06

**Authors:** Takashi Koike, Jingjing Sha, Yunpeng Bai, Yuhei Matsuda, Katsumi Hideshima, Takaya Yamada, Takahiro Kanno

**Affiliations:** 1 Department of Oral and Maxillofacial Surgery, Shimane University Faculty of Medicine, 89-1 Enya-Cho, Izumo, Shimane 693-8501, Japan; 2 Depart of Experimental Animals, Interdisciplinary Center for Science Research, Organization for Research, Shimane University, Izumo, Shimane 693-8501, Japan

**Keywords:** bacterial cellulose, bone morphogenetic protein-2, osteocalcin, proliferating cell nuclear antigen, sinus lift, bone regeneration

## Abstract

If the alveolar bone height of patients requiring dental implants in the maxillary molar region is inadequate, it is difficult to achieve satisfactory outcomes using existing bone graft materials. We previously reported the possible utility of bacterial cellulose (BC) as a new dental treatment material. BC has a high absorptive capacity, good mechanical strength, and good volume retention. BC loaded with bone morphogenetic protein-2 (BMP-2) might allow effective alveolar bone augmentation. We created critical frontal bone defect models in 12 male Japanese white rabbits and divided them into four groups: sham; BC (BC grafting only); BMP-2 (treated with BMP-2 solution only); and BC+BMP-2 (grafted with BC loaded with BMP-2). Newly formed bone volume was calculated via hematoxylin-eosin staining evaluation. The proliferating cell nuclear antigen and osteocalcin levels were determined by the immunohistochemical staining analysis. All measured indices of the BC+BMP-2 group were significantly superior to those of the other groups (all *p* < 0.05). BC maintained the graft space and released BMP-2 in a sustained manner, promoting optimal bone formation. The BC+BMP-2 combination enhanced bone regeneration and shows promise as a useful means of clinical pre-dental implant bone augmentation in the maxillary sinus.

## 1. Introduction

Although dental implantation is now a routine procedure, placing implants in the posterior maxillary region remains a challenge because the height of the alveolar process is often insufficient due to the location of the maxillary sinus. To resolve this anatomical constraint, Boyne and James [1,2,3] developed a technique for maxillary sinus floor elevation. The procedure involves grafting autogenous bone onto the sinus floor after opening the anterior wall of the maxillary sinus via the Caldwell–Luc procedure and separating and elevating the sinus floor membrane from the bone surface [1,2,3]. The prognosis for implant treatment with maxillary sinus floor elevation is good, and this method has been widely accepted as an essential technique for implantation in the posterior maxilla. Similarly, when implants are placed in a region with extensive bone loss or atrophy, autogenetic bone transplantation is the best method of treatment to avoid rejection [2,3,4,5,6,7,8]. Nevertheless, there are several limitations to this, including the invasive nature of the procedure to harvest bone and the limited amount of bone available to be collected, in addition to a long treatment duration, postoperative complications, and grafted bone resorption [9,10,11,12,13,14]. These limitations need to be resolved to make it possible to achieve more effective alveolar bone augmentation.

In the future, bone augmentation methods will need to incorporate alternative bone graft substitutes and enhance bone regeneration. Although various bone graft substitute methods have been explored, the outcomes have often been unsatisfactory because it is difficult to retain the bone’s three-dimensional structure [2,3,4,5,6,7]. The use of biocarriers to support regions where bone has been lost or atrophied is a promising alternative to autogenous bone grafts.

Bacterial cellulose (BC) is produced by certain strains of bacteria and differs substantially from plant cellulose. BC is a thin filamentous fiber approximately 1 μm in diameter that intertwines with itself and forms a crystalline, high-elasticity, and superabsorbent net-like structure. BC is sturdy, retains its shape, has a high-water content, and has many industrial applications in foodstuff preparation [15,16,17,18]. It has recently been used for skeletal and soft tissue grafting, to make artificial blood vessels, as a wound dressing, and to create ophthalmic scaffolds [19]. In terms of oral and maxillofacial surgical therapy, processed BC has been used for guided tissue regeneration in patients with periodontal disease [20]; we previously showed that BC has great potential when employed to treat dental root canals [15]. Patients who suffer trauma or develop bone diseases require guided bone regeneration, and biocompatible grafts are usually used to fill the defects and induce tissue regeneration. The excellent characteristics of BC suggest that the material may optimally facilitate dental bone regeneration prior to implantation.

Bone morphogenetic proteins (BMPs) are promising osteoinductive substances facilitating ectopic bone formation. When BMPs are loaded into a carrier and placed under muscle or skin, bone growth is induced, even in an area where there was no previous bone growth. This is achieved through enhanced differentiation of mesenchymal cells to osteoblasts and chondroblasts [8,21,22]. However, because BMPs easily diffuse from the carrier, it was previously thought impossible to maintain an effective concentration over a sufficiently long period to cause meaningful bone growth [8,23]. To increase the in vivo efficacy of BMP treatment, a carrier that can maintain its shape and allow controlled release is required [8,23]. Although type I collagen is effective, it does not maintain its three-dimensional structure for long enough [21,22,23,24]. BC has the properties required to be an effective carrier for BMPs. 

BMP-2 is the best-studied bone-forming protein. Currently, BMP-2 is the only bone morphogenetic protein approved as a bone graft substitute [25]. In oral and maxillofacial surgery, BMP-2 was approved by the United States Food and Drug Administration (FDA) in 2007 for sinus floor augmentation and extraction socket preservation [21,22]. Therefore, in this study, we utilized BC as a carrier for the sustained-release of BMP-2 in a rabbit frontal sinus model and performed histological and immunohistochemical staining to evaluate bone regeneration after maxillary sinus floor elevation. We aimed to confirm whether BC can serve as both a barrier membrane and a sustained-release drug carrier, maintaining space and promoting bone formation. Furthermore, we assessed whether the combination of BC and BMP-2 provide a useful means of clinical pre-dental implant bone augmentation in the maxillary sinus for the dental implants treatment.

## 2. Materials and Methods

### 2.1. Experimental Materials

#### 2.1.1. BC Sheets

**Figure 1:**
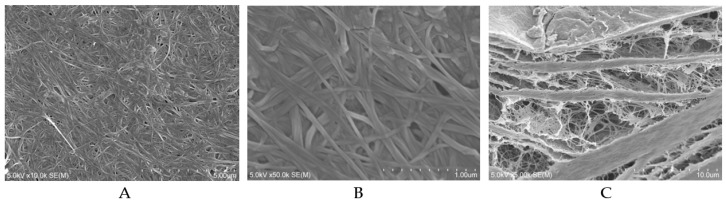
SEM micrographs of surface and cross-section of BC. (**A**) The surface of BC observed under 10.0 kx magnification. (**B**) The surface of BC observed under 50.0 kx magnification. (**C**) The cross-section of BC observed under 5000× magnification.

Two strains of *Acetobacter hansenii* (ATCC 700,178 and ATCC 35059, Sumisho Pharma International, Tokyo, Japan) were purchased from the American Type Culture Collection (Manassas, VA, USA). BC sheets (Department of Chemistry, Rikkyo University Faculty of Science, Tokyo, Japan) were obtained by culturing *Acetobacter hansenii* in 10-cm laboratory dishes containing CSL-Frc medium at 28 °C and a pressure of 101.3 kPa (20 vol% oxygen) for approximately three days until a 1-mm thick gel formed. The wet gel BC sheets were autoclaved three times for 20 min at 105 °C in deionized water, freeze-dried (FDU-1200, EYALA, Tokyo, Japan), and compressed to a thickness of 0.5 mm using a small press (J-1, AS ONE, Osaka, Japan) [15]. The compressed gel sheets were cut into 7 × 5 mm rectangles for later use. The ultrastructure of the BC detected by scanning electron microscopy (SEM) instruments (S-4800, Hitachi, Tokyo, Japan) was operated under 5.0 kV. The surface and cross-section morphology of the BC sample were shown in Figure 1. Briefly, porous interconnecting layers and compacting layers constitute a unique laminate structure within the BC. The coarse three-dimensional web of nanofibrils is interconnected with and superimposed upon each other, forming densely compact layers. The cellulose nanofibrils also constitute a uniform web-like structure on the surface of BC [15].

#### 2.1.2. BMP-2

A stock human-derived recombinant BMP-2 (rhBMP-2) solution (1.0 µg/µL) (Osteogenetics GmbH, Würzburg, Germany) was diluted to 0.5 μg/μL with sterilized distilled water (Otsuka distilled water, Otsuka Pharmaceutical, Tokyo, Japan) [26].

#### 2.1.3. BC+BMP-2 Composite

Ten microliters of rhBMP-2 solution (0.5 μg/μL) was carefully loaded onto the surface of each side of the BC sheet using a micropipette. Therefore, all BC sheets were loaded with 5 μg of rhBMP-2. As in the study by Silvestre et al. [27], SEM images showed that the drugs loaded in the BC could be uniformly distributed on the surface of the membrane. Furthermore, cross-sectional SEM analysis also demonstrated that the spaces inside the BC were filled with the drugs.

### 2.2. Experimental Method

Twelve male Japanese white rabbits (weight, 2.0–2.5 kg; age, 11 weeks) were purchased from BioTek (Saga, Japan). The animals were acclimated for one week at standard conditions (temperature, 23 ± 2 °C; humidity, 55 ± 10%; 12:12 h light/dark cycle) in a room that was ventilated at a rate of 13 times/h. They were fed ORC-4 (Oriental Yeast, Tokyo, Japan) and had free access to filtered water.

**Figure 2:**
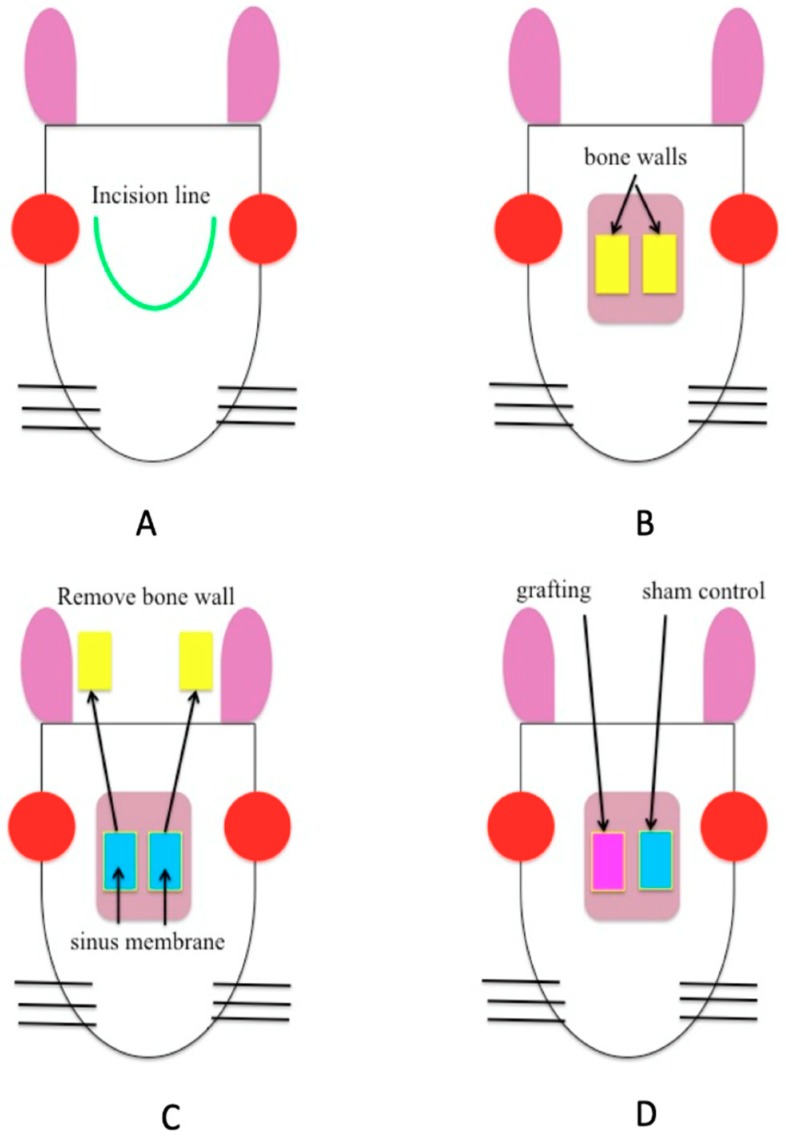
The schematics shows the experimental animal procedure of a rabbit frontal sinus model in the frontal view. Experimental animal procedure: (**A**). Incision line in the rabbit frontal sinus area; (**B**) Excision of 7 × 5 mm windows in the bilateral frontal bone; (**C**). Movement of the bone wall and reflection of the sinus membrane using a mucosal elevator to form sufficient grafting space; (**D**). The left side was used as a sham control (no graft) and the right side received the graft in the BC, BMP-2, and BC+BMP-2 groups.

The rabbits were anesthetized with 80 mg/kg ketamine HCl given intravenously (Ketalar, Sankyo, Tokyo, Japan) and 10 mg/kg xylazine was given intramuscularly in the rump (Selactar, Jin Kisara, Beyer, Tokyo, Japan). The fur over the surgical field was shaved, the skin was sterilized with 70% ethanol, and 0.3 mL of 2.0% lidocaine with 1/80,000 epinephrine (Xylocaine, Astellas Pharma, Tokyo, Japan) was infiltrated. Using a no. 11 surgical knife, a head arc incision extending under of periosteum was made and a skin periosteal flap was created (Figure 2A). The periosteum was protected from damage, and the frontal bone was exposed. A 7 × 5 mm window in the frontal bone was created bilaterally using a dental fissure bar (Figure 2B). After removing the bone, the sinus membrane was reflected using a mucosal elevator to form a space (Figure 2C). The left side served as the sham area and the right side the experimental area (Figure 2D). The rabbits were divided into four groups: sham; BC (BC alone was grafted); BMP-2 (rabbits were given only 10 μL BMP-2 (0.5 μg/μL)); and BC+BMP-2 (grafted with BC loaded with 10 μL BMP-2 (0.5 μg/μL)). After placing the graft, the bone wall was covered and the periosteum and skin were sutured. The rabbits awoke 1–2 h after the operation and exhibited normal behavior and appetite.

After grafting, no surgical complications occurred and no visual signs of inflammation or infection were observed. The four rabbits from each group were sacrificed at four and eight weeks by injecting 40 mg/kg pentobarbital sodium into a large auricular vein (Nembutal, Dainippon Sumitomo Pharma, Osaka, Japan). A thoracoabdominal skin incision was made and the sternum was removed to expose the heart. The left ventricle was cannulated, and the right atrium was cut open. Reflux blood removal was performed with saline containing heparin.

All animal experiments followed the guidelines for humane care and use of laboratory animals of the Shimane University Faculty of Medicine, and the study protocol was approved by our Institutional Animal Ethics Committee (approval number IZ 27-46).

### 2.3. Hematoxylin–Eosin (H and E) Staining

The specimens were fixed in 20% phosphate-buffered formalin solution (MUTO PURE CHEMICALS CO., LTD, Tokyo, Japan), decalcified with ethylenediaminetetraacetic acid (EDTA), embedded in paraffin, and cut with a microtome (LEICA JUNG HISTOSLIDE 2000R, Leica Instruments GmbH, Wetzlar, Germany) into 5-μm-thick slices. All slices were stained with hematoxylin solution (MUTO PURE CHEMICALS CO., LTD, Tokyo, Japan) for 5 min followed by five dips in 1% (*v*/*v*) acid ethanol (1% HCl in 70% ethanol, both *v*/*v*) and then rinsed in distilled water. Next, the sections were stained with eosin solution (MUTO PURE CHEMICALS CO., LTD, Tokyo, Japan) for 3 min, followed by dehydration in graded alcohol (MUTO PURE CHEMICALS CO., LTD, Tokyo, Japan) baths and clearing in xylene (NACALAI TESQUE, INC., Kyoto, Japan). The slides were finally examined under a light microscope (BX43, OLYMPUS, Tokyo, Japan). (MUTO PURE CHEMICALS CO., LTD, Tokyo, Japan)

### 2.4. Immunohistochemical Staining

The tissue slices were also subjected to immunohistochemical staining with primary mouse anti-osteocalcin (anti-OC) monoclonal antibody (1:3000; Abcam, Cambridge, UK) and primary mouse anti-proliferating cell nuclear antigen (anti-PCNA) monoclonal antibody (1:800; Dako, Tokyo, Japan) to examine osteoblast differentiation and cell proliferation. Sections were deparaffinized with xylene substituted for ethanol for hydrophilization, and antigens were activated in an autoclave (HIGH-PRESSURE STEAM STERILIZER LBS-325, TOMY SEIKO CO., LTD, Tokyo, Japan) (at 80 °C for 8 min) using citrate buffer and distilled water before annealing to room temperature over a 12-h period. The specimens were incubated overnight at 25 °C with mouse anti-OC monoclonal antibody and mouse anti-PCNA monoclonal antibody as primary antibodies. After incubation, the secondary antibody was added at 25 °C and reacted with an EnVision kit (Dako, Tokyo, Japan) for 1 h. The color was intensified using DAB solution (Dako, Tokyo, Japan) and counterstained with hematoxylin. The slices were then observed under a light microscope (BX43, OLYMPUS, Tokyo, Japan).

### 2.5. Histomorphometric Examination

Tissue images were obtained using a microscope at 20× magnification. A digital image of the entire bone defect was constructed by integrating the images. All images were analyzed with the aid of ImageJ software (Image J 2.0.0-rc-43/1.50e, NIH, Bethesda, MD, USA) and the extent of newly formed bone was estimated as the ratio of the area of newly formed bone to that of the entire bone defect as revealed by H and E staining [28,29].

After immunohistochemical staining for PCNA and OC, we photographed all slices at 40× magnification. Three visual fields were selected in the (randomly chosen) upper, lower, right, or left quadrants surrounding the grafts in the BC and BC+BMP-2 groups. For the BMP-2 and sham groups, we randomly selected three areas between the bone wall and sinus membrane. We calculated the PCNA-positive cell ratio of target cells to the entire number of cells within the visual field. The target cells were osteoblasts for estimating bone formation and fibroblasts for estimating wound healing at the bone augmentation area; inflammation-related cells were excluded. The OC-stained area ratio was the ratio of the stained area to the total area.

### 2.6. Statistical Analysis

We used a one-way analysis of variance and the LSD-*t* test to compare the newly formed bone values, the PCNA-positive cell ratios, and the OC-stained area ratios among groups. Independent-samples *t*-tests were used to compare the newly formed bone values, PCNA-positive cell ratios, and OC-stained area ratios among different time points. All statistical analyses were performed using SPSS ver. 24.0 (IBM, Armonk, NY, USA). A *p*-value < 0.05 indicated statistical significance.

## 3. Results

### 3.1. H and E Staining Evaluation

#### 3.1.1. Histomorphology

**Figure 3:**
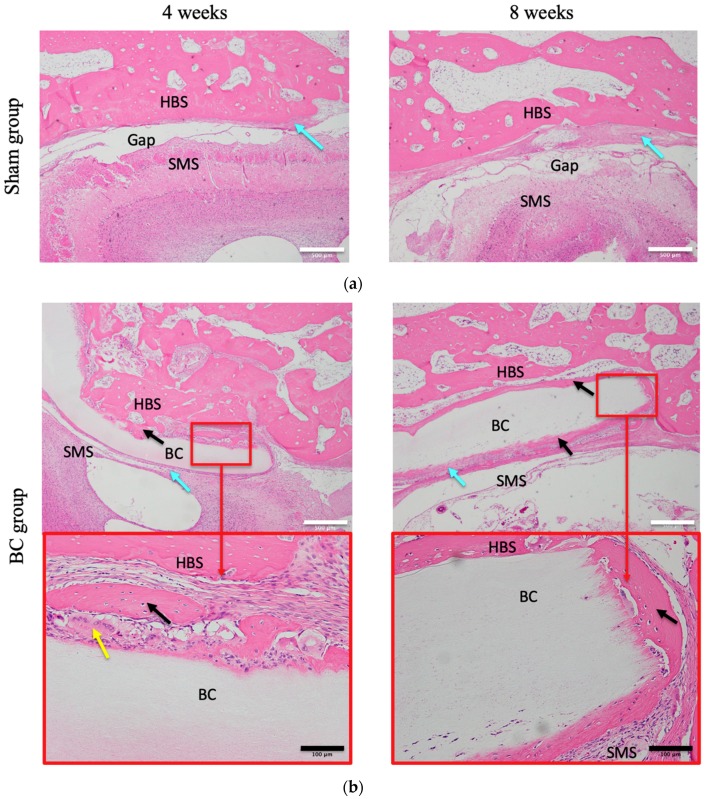

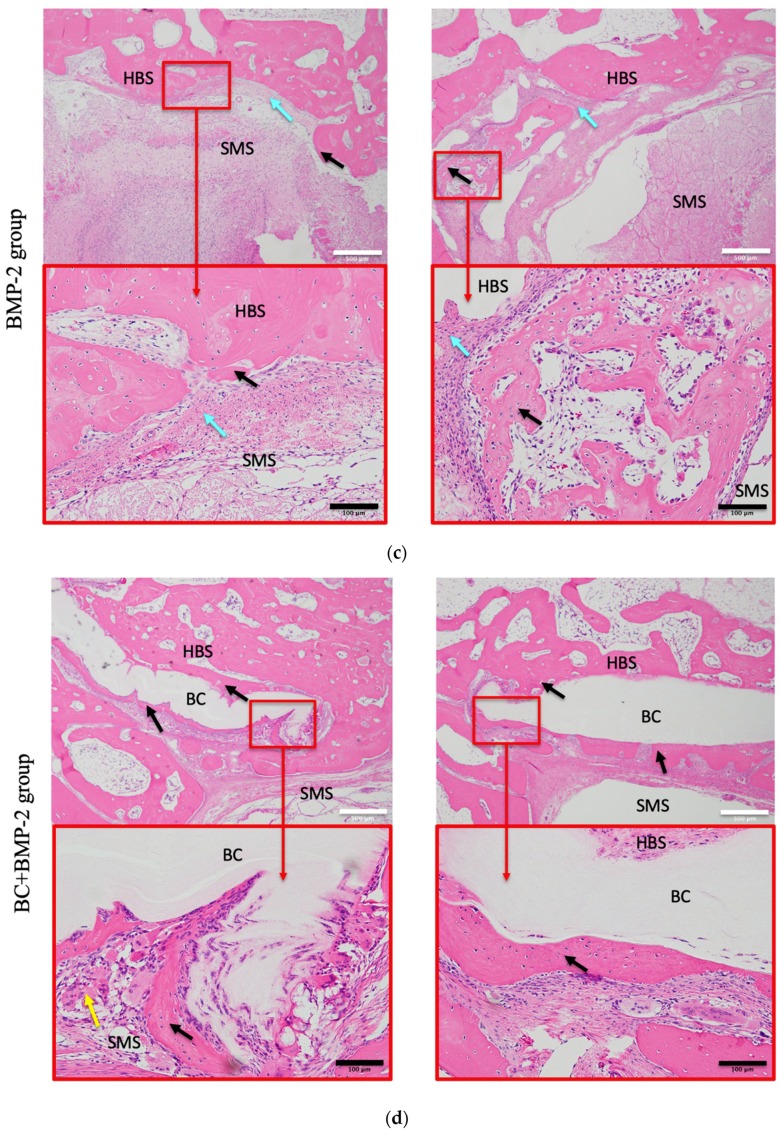
H&E staining in the (**a**): sham (n = 3), (**b**): BC (n = 3), (**c**): BMP-2 (n = 3), and (**d**): BC+BMP-2 (n = 3) groups at four and eight weeks. Blue arrows indicate connective tissue, black arrows indicate newly formed bone, and yellow arrows indicate cartilage tissue. HBS, host bone side; SMS, sinus membrane side; BC, bacterial cellulose. White scale bars (500 μm) indicate slices at 4× magnification; black scale bars (100 μm) indicate slices at 20× magnification.

To evaluate bone formation after grafting, decalcified sections of frontal critical bone defects were subjected to H&E staining. No inflammatory reaction was observed at any time in any group. Either no, or only a small amount of, regenerated (newly formed) osteoid tissue was observed between the frontal bone wall and the sinus membrane in the sham group at any time: clear gaps were evident between the wall and membrane. The extent of new bone growth in the BMP-2 group was slightly greater than that in the sham group: gaps in the connective tissue and newly formed bone were filled gradually. In the BC group, connective and fibrous tissue filled gaps between the BC and sinus membrane at four weeks, and unmineralized new bone and cartilage-like tissue were evident between the bone wall and the BC. However, at eight weeks, regardless of the side, the newly formed bone obviously surrounded the BC. In the BC+BMP-2 group, newly formed bone and cartilage-like tissue surrounded the BC, and clumps of chondrocytes and osteoblasts invaded the BC+BMP-2 composite at four weeks. At eight weeks, abundant mature calcified new bone bonded strongly to the BC+BMP-2 sheet. Bone lacunae hosting mature osteocytes were evident in the mature calcified new bone. Notably, at eight weeks, the extent of new mature bone growth was substantially greater than that in any other group at any time (Figure 3).

#### 3.1.2. H and E Staining of Newly Formed Bone

**Figure 4:**
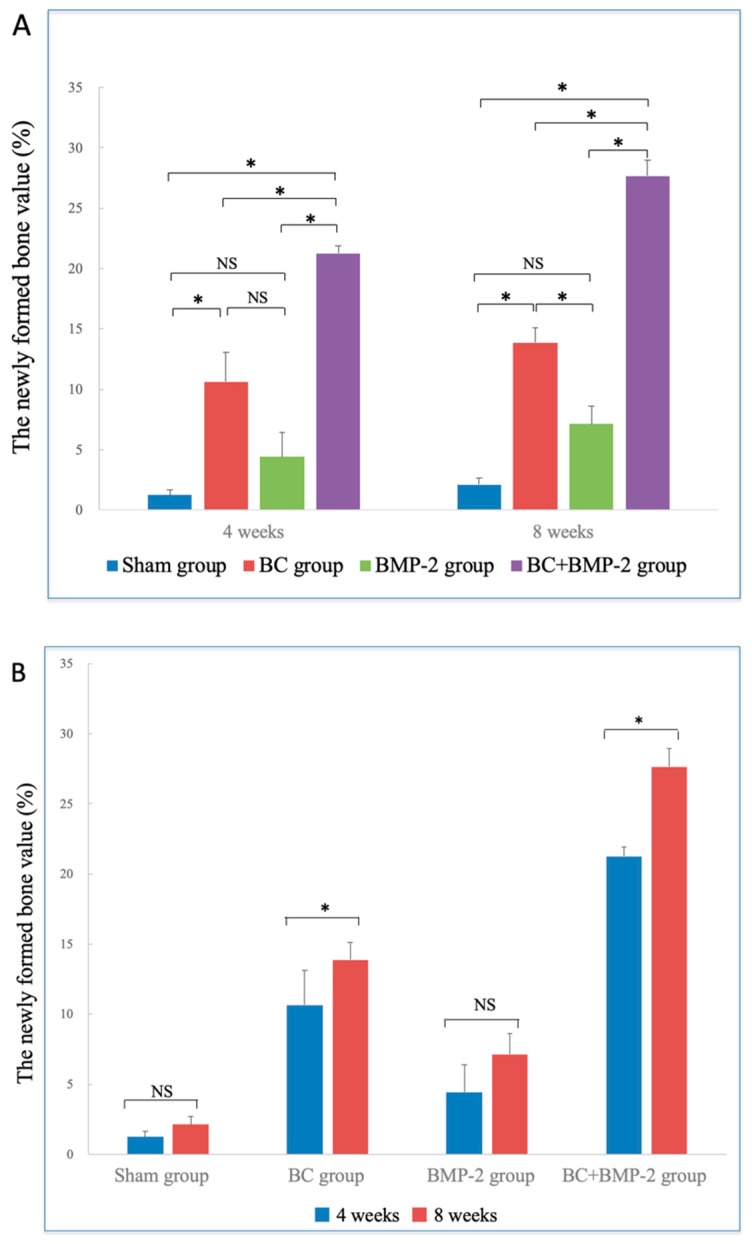
H&E staining analysis of newly formed bone values: (**A**) Comparison of newly formed bone values between different groups, analyzed by One-way analysis of variance and LSD-*t* test. * *p* < 0.05; NS: no significance. Error bars indicate standard deviations. (**B**) Comparison of newly formed bone values between four and eight weeks, analyzed by independent-samples *t*-test; * *p* < 0.05; NS: no significance. Error bars indicate standard deviations.

The extent of newly formed bone in the BC+BMP-2 group was much greater than that in any other groups at both four and eight weeks (all *p* < 0.05). Additionally, the quality of newly formed bone in the BC group was superior to that in the BMP-2 group; BC was more significant in terms of osteogenesis than was BMP-2. However, no significant difference was apparent between the BMP-2 and the sham groups (*p* > 0.05), indicating that BMP-2 alone did not significantly enhance osteogenesis (Figure 4A). At eight weeks, the extent of newly formed bone in all groups was clearly greater than at four weeks, especially in the BC+BMP-2 group (*p* < 0.05) (Figure 4B).

### 3.2. Immunohistochemical Evaluation

#### 3.2.1. PCNA

**Figure 5:**
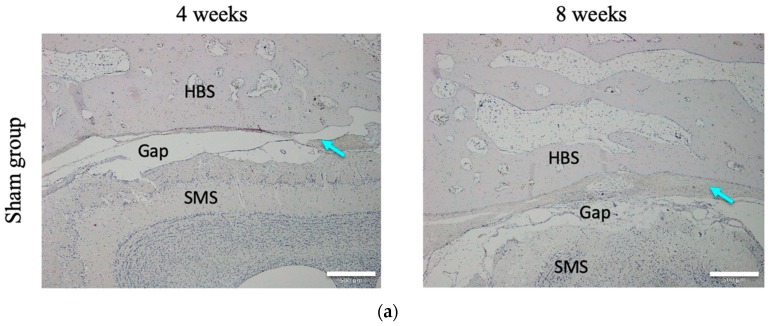

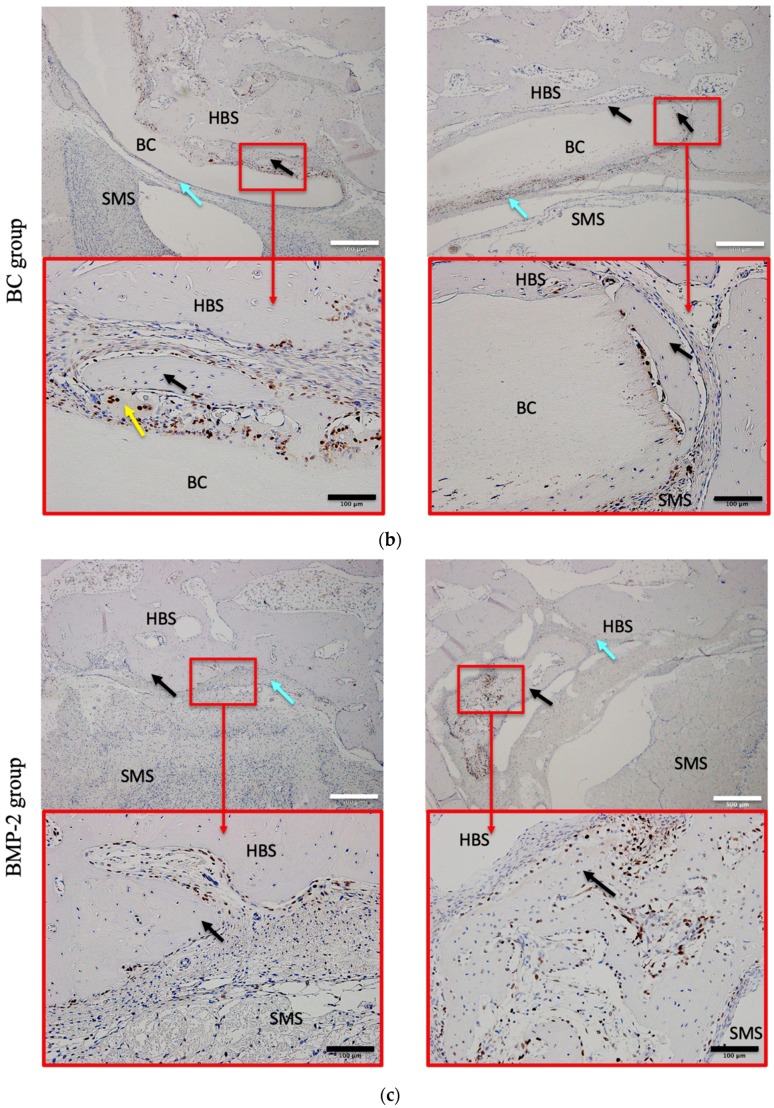

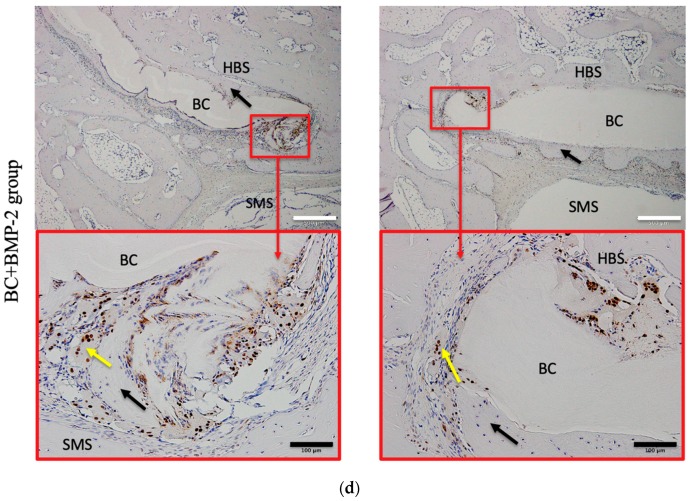
Immunohistochemical staining of PCNA expression in the (**a**): sham (n = 3), (**b**): BC (n = 3), (**c**): BMP-2 (n = 3), and (**d**): BC+BMP-2 (n = 3) groups at four and eight weeks. Blue arrows indicate connective tissue, black arrows indicate newly formed bone, and yellow arrows indicate cartilage tissue. HBS, host bone side; SMS, sinus membrane side; BC, bacterial cellulose. White scale bars (500 μm) indicate slices at 4× magnification; black scale bars (100 μm) indicate slices at 20× magnification.

**Figure 6:**
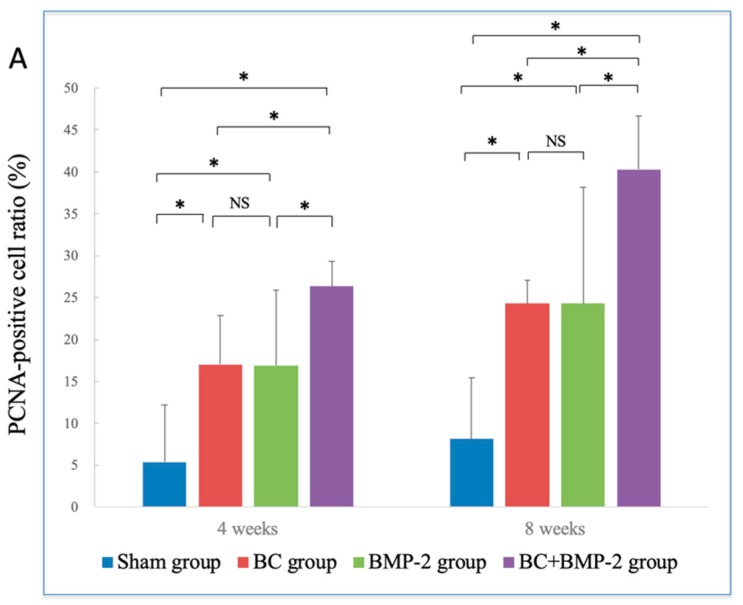

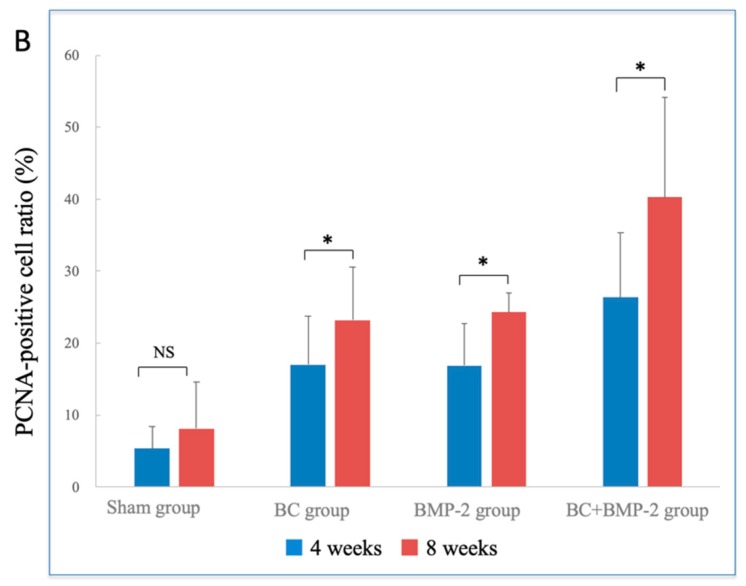
Immunohistochemical staining analysis of PCNA-positive cell ratios: (**A**) Comparison of the PCNA-positive cell ratios between groups, analyzed by one-way analysis of variance and LSD-*t* test. * *p* < 0.05; NS: no significance. Error bars indicate standard deviations. (**B**) Comparison of PCNA-positive cell ratios between time points, analyzed by independent-samples *t*-test. * *p* < 0.05; NS: no significance. Error bars indicate standard deviations.

PCNA-positive cells (which stained dark brown) were detected in all sections (Figure 5). However, in the experimental groups, the positivity rates were significantly higher than in the sham group (*p* < 0.05, Figure 6A). In all groups, PCNA accumulated around newly formed bone and tissue adjacent to the periosteum at both four and eight weeks. At four weeks, very few PCNA-positive cells were observed near the host bone in the sham group. However, large numbers of such cells were apparent in the BC and BMP-2 groups, and were spread much more extensively within the region between the host bone and the frontal sinus membrane. In the BC group, PCNA-positive cells were abundant around the BC; the number was far greater than on the sham side. However, the PCNA-positive cell distribution pattern in the BMP-2 group differed from that in the BC group; most cells lay close to the host bone, not in the stromal area. This difference did not attain statistical significance (*p* > 0.05, Figure 6A). The distribution pattern in the BC+BMP-2 group was similar to that in the BC group, but much more prominent. Over the first four weeks, the BC+BMP-2 group exhibited a dramatic increase in the number of PCNA-positive cells compared to the other groups (*p* < 0.05, Figure 6A). Many positive cells accumulated around the BC+BMP-2 composite, where newly formed bone was also evident. Inside new bone, the chondrocytes were positive for PCNA, as were the fibroblasts and osteoblasts.

Similar trends were apparent at eight weeks. Unlike the sham group, the BC, BMP-2, and BC+BMP-2 groups all exhibited significant improvements compared to four weeks (*p* < 0.05, Figure 6B). As healing progressed, the space between the host bone and the sinus membrane narrowed. In the latter three groups, the numbers of PCNA-positive cells in the stroma increased dramatically by eight weeks compared to four weeks (*p* < 0.05, Figure 6B). In the BC+BMP-2 group, newly formed bone was obvious, much more so than in the BC and BMP-2 groups. The cells surrounded the BC+BMP-2 composite, and new bone contained many more PCNA-positive cells than did regions distant from the composite.

#### 3.2.2. OC

**Figure 7:**
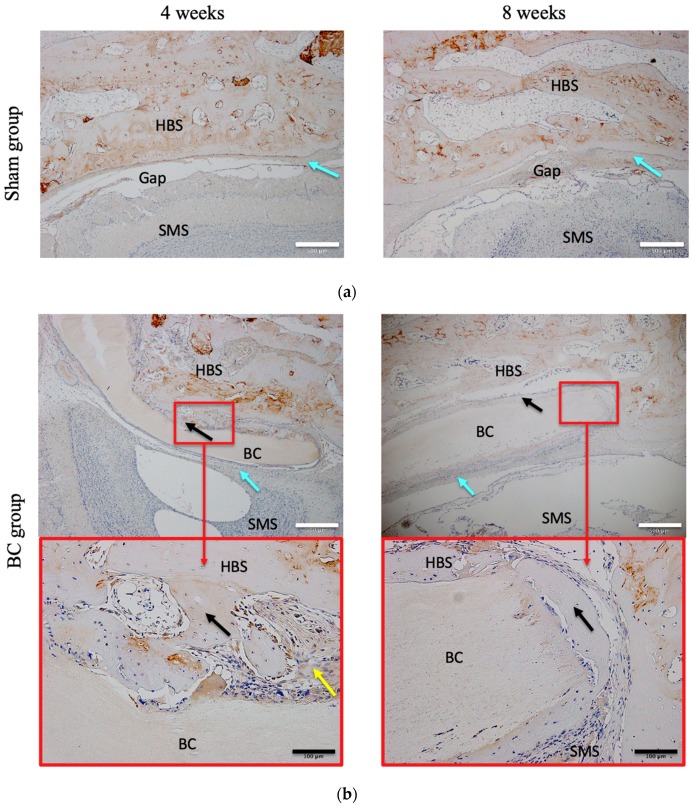

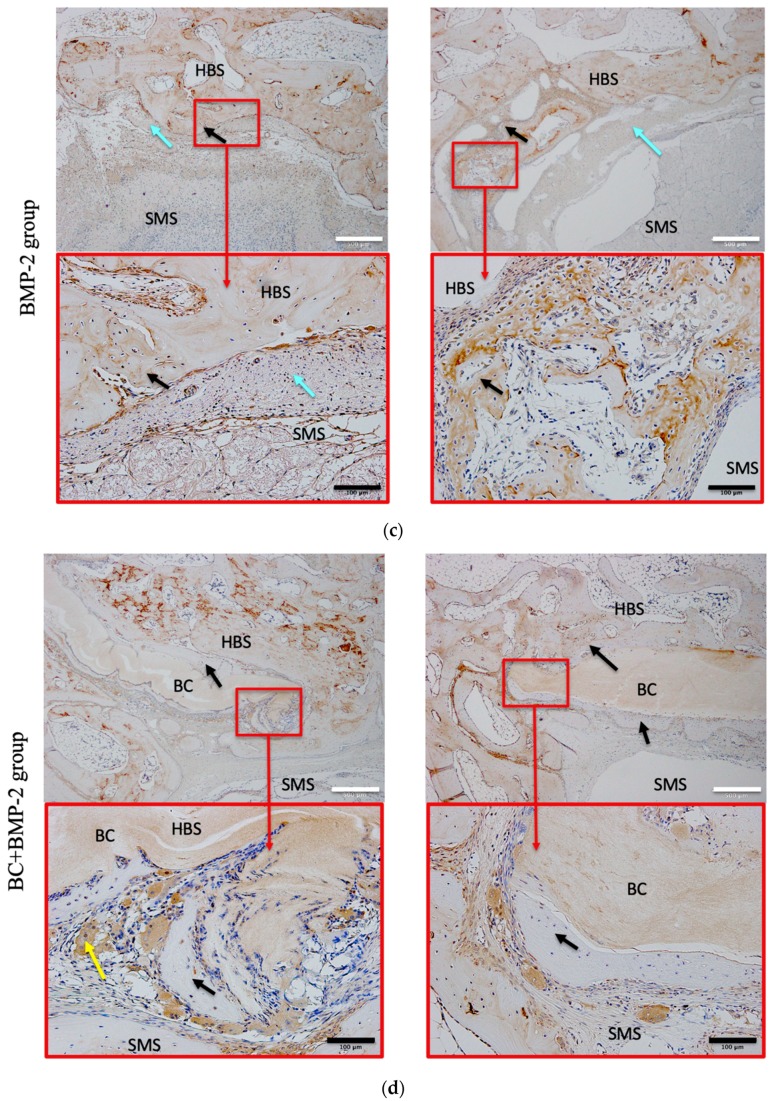
Immunohistochemical staining of OC expression in the (**a**): sham (n = 3), (**b**): BC (n = 3), (**c**): BMP-2 (n = 3), and (**d**): BC+BMP-2 (n = 3) groups at four and eight weeks. Blue arrows indicate connective tissue, black arrows indicate newly formed bone, and yellow arrows indicate cartilage tissue. HBS, host bone side; SMS, sinus membrane side; BC, bacterial cellulose. White scale bars (500 μm) indicate slices at 4× magnification; black scale bars (100 μm) indicate slices at 20× magnification.

**Figure 8:**
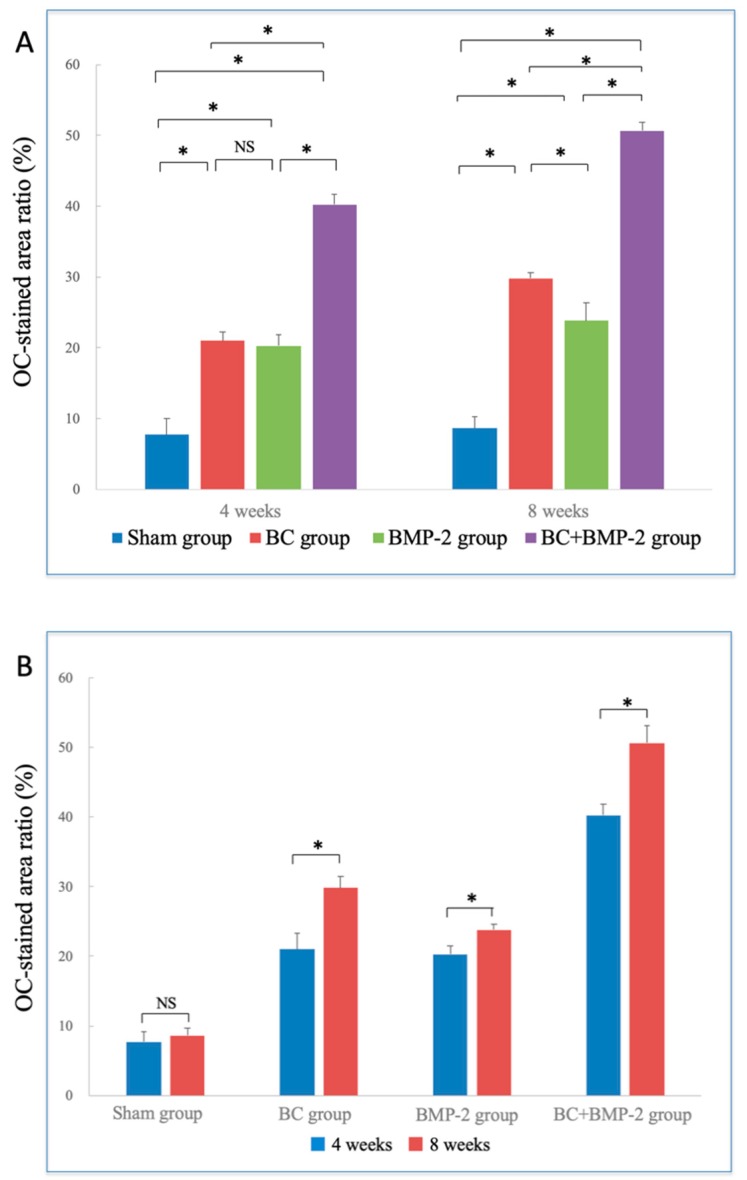
Immunohistochemical staining analysis of OC-stained area ratios: (**A**) Comparison of OC-stained area ratios between groups, analyzed by one-way analysis of variance and LSD-*t* test. * *p* < 0.05; NS: no significance. Error bars indicate standard deviations. (**B**) Comparison of OC-stained area ratios between time points, analyzed by independent-samples *t*-test. * *p* < 0.05; NS: no significance. Error bars indicate standard deviations.

Figure 7 presents OC-stained specimens. OC was expressed principally in the extracellular matrix (staining yellow or brown, Figure 7). At four weeks, the BC+BMP-2 group had the highest OC-positive area ratio of all four groups (*p* < 0.05, Figure 8A). In the sham group, the positive area lay largely adjacent to the host bone. No obvious staining was evident in the stroma, and any staining that was observed was very weak. Both the staining intensity and area were greater in the BC and BMP-2 groups than the sham group. In the BC group, the OC-positive area was not confined to regions close to the host bone, but also surrounded the BC; a few small areas of newly formed bone were also apparent. Compared to the BC group, the OC pattern in the BMP-2 group was similar to that of the sham group, but much stronger. Most positive regions lay close to the host bone; very few newly formed bone-like regions were scattered among the stroma, and these expressed OC. The BC and BMP-2 groups did not differ at four weeks (*p* > 0.05, Figure 8A). At this time, the BC+BMP-2 group had a significant increase in bone formation compared to the other groups. Osteoblasts accumulated around the BC+BMP-2 composite, and positively stained cells and areas were very apparent.

At eight weeks, all experimental groups (not the sham group) evidenced larger OC-positive areas than at four weeks (*p* < 0.05, Figure 8B); in the sham group, the extent of OC expression had barely changed. In the BC and BMP-2 groups, the staining intensity and area were both significantly greater than at four weeks (both *p* < 0.05, Figure 8B). OC-positive cells scattered in the stroma of both groups were much easier to detect than at four weeks, but the BC group had a much greater positive area than the BMP-2 group (*p* < 0.05, Figure 8B). The BC+BMP-2 group differed significantly from all other groups at both four and eight weeks (*p* < 0.05, Figure 8B). Newly formed bone of larger area was very easily detected; the positively stained area contained bone matrix and osteoblasts, as well as some chondrocytes. All positive staining lay adjacent to the BC+BMP-2 composite.

## 4. Discussion

During dental implant placement, the membrane barrier technique is generally employed. Particularly in the molar regions, the anatomical structure of the maxillary sinus dictates that the membrane technique must always be combined with sinus floor elevation to provide a safe, secluded, and sufficient space for implants. Several non-resorbable and bioresorbable barrier membranes have been used. Such devices must manifest biocompatibility and stability over the time required for barrier integrity, space maintenance, exclusion of undesirable cell ingrowth, and ease of use [30,31,32]. Of the available non-resorbable membranes, cellulose-based membranes have been widely used during implant or tissue regeneration surgery [19,20]. We previously showed that BC exhibited great potential for treatment of dental root canals: it can serve as both a carrier and a barrier, sustainably releasing drugs and maintaining the necessary space [15]. During guided bone regeneration treatment, patients who have suffered trauma or have a bone disease usually require biocompatible grafts that fill defects and induce bone regeneration. BC may be invaluable in these contexts. Thus, we evaluated the use of BC as a barrier by grafting BC into the frontal sinus, with and without BMP-2.

No rabbit exhibited obvious inflammation (redness, exudation) at either four or eight weeks. No histological sign of chronic inflammation was apparent. No capsule formation developed around BC or the BC+BMP-2 composite. No foreign body reaction was detected in tissue around BC. When evaluating the long-term outcomes of an implanted scaffold, it is essential to exclude initiation of a chronic inflammatory response. We found that BC exhibited excellent biocompatibility to eight weeks after placement, these results are also consistent with previous studies [19].

In the BC-only group, at four weeks, connective tissue and fibrous tissue were present around the BC. However, at eight weeks, the tissue was much more mature and organized, featuring a small amount of newly formed ectopic bone tissue surrounding the BC (Figure 3). This healing pattern resembles normal healing: stem cells accumulate and ingrow, fibroblasts accumulate and become organized into mature connective tissue, and ossification proceeds. At eight weeks, the BC+BMP-2 became well-integrated with rabbit tissue (Figure 3). Such excellent biocompatibility deserves further study. It is also important to investigate whether new bone formation is induced by BC per se, or attributable only to the three-dimensional space provided by BC. The BC used was that of *Acetobacter hansenii*; during biosynthesis, BC forms a pellicle containing a random microfibrillar network of cellulose chains interspersed with amorphous regions [33]. BC contains both type I cellulose (parallel chains) and type II cellulose (anti-parallel chains) stabilized by intimate hydrogen bonding, forming a low-energy three-dimensional structure that is much more stable than cellulose type I [33,34]. The internal randomly oriented nanofibers impart a large surface area to BC. This internal architecture endows BC with unique properties including a high tensile strength, high-level crystallinity, and a high water-holding capacity [35]. BC is an optimal three-dimensional substrate for cell attachment; the micro-fibrillar structure is associated with flexibility and good gas exchange [36]. A grafted BC membrane serves as a physical barrier reducing pain and the risk of infection, and allows drug transfer to the wound [37]. Thus, when combined with BMP-2, BC creates a non-inflammatory environment facilitating excellent bone regeneration.

Humans encode 20 BMPs; after birth, BMPs are associated with the collagenous extracellular matrix, being expressed by both periosteal and mesenchymal cells of the marrow stroma during fracture repair [38,39]. The BMP family is divided into four distinct subfamilies, and BMP-2 and BMP-4 are members of the first family [40]. BMP-2 has the ability to induce bone tissue growth, even at sites where no bone was present originally [21,22,23]. BMP-2 can exert pleiotropic effects on the various steps of bone morphogenesis, depending on BMP-2 concentration [41]; higher concentrations promote mitogenesis and differentiation [40]. However, rapid diffusion, degradation, and/or absorption of extraneous BMP-2 delivered in vivo are major concerns, as revealed by our BMP-2-only group data. Only very low levels of newly formed cartilage and bone were observed (Figure 3). An excellent slow-release carrier is required to allow BMP-2 to exert its full potential in terms of inducing bone growth. A previous study compared potential carriers including collagens, tricalcium phosphate, and hydroxyapatite, and found that insoluble collagen and hydroxyapatite were optimal [40]. BC may thus serve as an optimal BMP-2 carrier. BC is insoluble in water, and cellulose degradation by animals and humans is limited because of the absence of hydrolases attacking the β (1–4) glycosidic linkages of BC [42]. We found that the extent of new bone formation was greatest in the BC+BMP-2 group, followed by the BC and the BMP-2 groups, at both four and eight weeks (Figure 3). Thus, BC may be a very useful carrier during bone regeneration.

PCNA and OC status was used to evaluate bone regeneration and cell viability. PCNA is a nucleoprotein of molecular weight of 36 kDa expressed from late G1 to S phase [43]. PCNA is thus a reliable marker of cell proliferation. Although PCNA-positive cells were found in all groups, the differences between the sham and experimental groups were marked (Figure 5). The BC+BMP-2 group expressed many more PCNA-positive cells than any other group at both four and eight weeks. We also measured OC expression. OC is a non-collagenous protein in bone consisting of 49 amino acids with a molecular weight of 58 kDa [44,45] and serves as a marker of bone formation, because it can be observed during the late differentiation phase of osteoblasts. Compared with the sham group, OC was obviously upregulated in the experimental groups (Figure 7). The OC-positive region was significantly larger in the BC+BMP-2 group than the other two experimental groups (Figure 7). The OC-positive area of the BC group was somewhat larger than that of the BMP-2 group, but statistical significance was not attained.

A larger OC-positive area was associated with higher PCNA-positivity in all experimental groups, especially in the BC+BMP-2 group. PCNA is not an independent prognostic indicator of cell activity; rather (using the analogy of Maga et al. [46]), PCNA is a “dancer” with many “partners”. When PCNA associates with OC, the combination reveals high-level cell proliferation and differentiation. OC is secreted principally by mature osteoblasts, and PCNA was originally identified as an antigen expressed during the DNA synthesis phase of the cell cycle [42]. Increases in both PCNA and OC levels enhance bone tissue regeneration and remodeling.

Notably, the PCNA and OC expression trends were highly consistent with our histomorphological data. We predicted that new bone formation would commence relatively early. It appears that a sustained-release of BMP-2 is effective for enhancing osteogenic cell differentiation, and thus increasing the formation of new bone. Both the BC and BMP-2 groups had more osteoblast differentiation and proliferation than the sham control, but the two groups were similar in these contexts especially at an early bone healing stage. Thus, both a physical structure and chemical stimulation are essential to promote healing. After BC was loaded with BMP-2, healing and bone regeneration were remarkably accelerated. BMP-2 is known to stimulate bone regeneration. However, further study is needed to investigate whether BMP-2 loaded into BC stimulates tissue healing in a manner similar to BMP-2 alone.

In our previous study, we tested the drug release of BC by loading with saline, K^+^-free electrolyte fluid, and electrolyte fluid. Cumulative release of trypan blue revealed that, within the initial 3 min, the drug was released rapidly and achieved a comparatively high level at 6 h; subsequently, the release rate tended to be stable and lasted more than seven days. No significant difference in modulus for BC was found between six hours and seven days [15]. The time-dependent drug release profile of BMP-2 was presumed to be similar to that of our previous result. Other studies suggested that the drug release rate in BC composite is influenced by several factors, such as BC film thickness, body temperature, and pH conditions; these variables affect swelling of the nanofibers and the porosity of the material [27]. Freire and Silvestre’s groups systematically studied the utilization of BC membranes for transdermal delivery of a series of drugs and found that the maximum release was achieved within 1 h [47,48]. However, if the pH conditions changed, as in the study of Huang et al., the drug-release profile was altered dramatically [49]. Nevertheless, it remains unclear whether the release profile of subcutaneous and intraosseous BC composite remains similar; additional studies are needed in this field.

By varying the bacterial culture time, it is possible to produce BC of various fibril densities, which are associated with different properties appropriate in particular circumstances [50]. In previous studies on wound-healing, BC membranes facilitated rapid epithelialization and tissue regeneration of even second- or third-degree burns; BC membranes sustained moisture levels and absorbed exudates [51]. BC sheets also serve as excellent tympanic membrane grafts for patients with local perforations [52,53].

BC is a versatile biomaterial exhibiting excellent biocompatibility, and can integrate with host tissue without provoking chronic inflammation. These properties were well-demonstrated in our present study. However, this study spanned only eight weeks, so more research is needed to investigate how bone derived by grafting changes over time. It will also be necessary to examine concentration-dependent bone formation by BMP-2 in order to find the most effective dose [8,26]. Manipulating the composition of BC could generate new material that is even safer and more effective in the long term. Moreover, further clinical study may involve higher animals, such as dogs or monkeys [54,55]. We believe that BC has a bright future, and will serve as an excellent biomaterial for tissue engineering.

## 5. Conclusions

The histological and immunohistochemical evaluations of the present study confirmed the excellent biocompatibility of BC in a rabbit frontal sinus model. Combined with BMP-2, BC allowed excellent bone regeneration. The results suggest that BC can serve as both a barrier membrane and a sustained-release drug carrier, maintaining space and promoting bone re-formation. BC is an ideal biomaterial for dental implant surgery, facilitating sinus membrane elevation and exhibiting other clinical utilities.
